# Ethnobotany and Antimicrobial Peptides From Plants of the Solanaceae Family: An Update and Future Prospects

**DOI:** 10.3389/fphar.2020.00565

**Published:** 2020-05-07

**Authors:** Mohasana Afroz, Sanzida Akter, Asif Ahmed, Razina Rouf, Jamil A. Shilpi, Evelin Tiralongo, Satyajit D. Sarker, Ulf Göransson, Shaikh Jamal Uddin

**Affiliations:** ^1^Pharmacy Discipline, Life Science School, Khulna University, Khulna, Bangladesh; ^2^Biotechnology and Genetic Engineering Discipline, Life Science School, Khulna University, Khulna, Bangladesh; ^3^Department of Pharmacy, Faculty of Life Science, Bangabandhu Sheikh Mujibur Rahman Science & Technology University, Gopalganj, Bangladesh; ^4^School of Pharmacy and Pharmacology, Griffith University, Southport, QLD, Australia; ^5^Centre for Natural Products Discovery, School of Pharmacy and Biomolecular Sciences, Liverpool John Moores University, Liverpool, United Kingdom; ^6^Biomedical Center, Division of Pharmacognosy, Uppsala University, Uppsala, Sweden; ^7^Biomedical Center, Department of Medicinal Chemistry, Uppsala University, Uppsala, Sweden

**Keywords:** antimicrobial peptides, Solanaceae, ethnobotany, antibiotic resistance, traditional medicine

## Abstract

The Solanaceae is an important plant family that has been playing an essential role in traditional medicine and human nutrition. Members of the Solanaceae are rich in bioactive metabolites and have been used by different tribes around the world for ages. Antimicrobial peptides (AMPs) from plants have drawn great interest in recent years and raised new hope for developing new antimicrobial agents for meeting the challenges of antibiotic resistance. This review aims to summarize the reported AMPs from plants of the Solanaceae with possible molecular mechanisms of action as well as to correlate their traditional uses with reported antimicrobial actions of the peptides. A systematic literature study was conducted using different databases until August 2019 based on the inclusion and exclusion criteria. According to literature, a variety of AMPs including defensins, protease inhibitor, lectins, thionin-like peptides, vicilin-like peptides, and snaking were isolated from plants of the Solanaceae and were involved in their defense mechanism. These peptides exhibited significant antibacterial, antifungal and antiviral activity against organisms for both plant and human host. *Brugmansia, Capsicum*, *Datura*, *Nicotiana, Salpichora, Solanum*, *Petunia*, and *Withania* are the most commonly studied genera for AMPs. Among these genera, *Capsicum* and the *Solanum* ranked top according to the total number of studies (35%–38% studies) for different AMPs. The mechanisms of action of the reported AMPs from Solanaceae was not any new rather similar to other reported AMPs including alteration of membrane potential and permeability, membrane pore formation, and cell aggregation. Whereas, induction of cell membrane permiabilization, inhibition of germination and alteration of hyphal growth were reported as mechanisms of antifungal activity. Plants of the Solanaceae have been used traditionally as antimicrobial, insecticidal, and antiinfectious agents, and as poisons. The reported AMPs from the Solanaceae are the products of chemical shields to protect plants from microorganisms and pests which unfold an obvious link with their traditional medicinal use. In summary, it is evident that AMPs from this family possess considerable antimicrobial activity against a wide range of bacterial and fungal pathogens and can be regarded as a potential source for lead molecules to develop new antimicrobial agents.

## Introduction

Misuse or overuse of antibiotics is now becoming the major contributing factor for the ever-increasing antimicrobial resistance (Chandra et al., 2017). Discovery of new effective antimicrobial agents has become a dire need to combat antibiotic resistance which is posing as one of the biggest threat to global health. Since ancient time, natural products have been playing an essential role around the world to treat human diseases as well as a potential source of new therapeutic agents because of their unique and immense chemical diversity (Amedeo Amedei and Niccolai., 2014). Ethnopharmacology, a multidisciplinary study of indigenous remedies, has a great significance on discovery of new drug from natural sources (Holmstedt and Bruhn, 1983).

It is well known that plants can develop different constitutive and inducible mechanisms for the protection from pathogenic infection *via* morphological barriers, secondary metabolites or antimicrobial peptides (AMPs) (Benko-Iseppon et al., 2010). AMPs belong to a wide range of protein family that act as a part of innate immune system or barrier defense of all higher living organisms (Broekaert et al., 1997; Hancock, 2001; Diamond et al., 2009). In recent years, AMPs are getting interest as a surrogate of conventional antibiotics because of their significant activity against multidrug resistant organisms by their direct action on microorganisms or stimulating immune responses (Marshall and Arenas, 2003; Pushpanathan et al., 2013; Mahlapuu et al., 2016). Natural AMPs are reported to possess low to no toxicity in humans and are stable in various conditions because of their unique features including disulfide bonds, overall charges, and especial structural conformation (Barbosa Pelegrini et al., 2011; Bondaryk et al., 2017). Exceptional features of AMPs make them potential candidate to develop new antimicrobial agents. About 1,500 AMPs have been identified from natural sources and a number of these are presently under clinical or preclinical trials (e.g. kalata B1 and B2, pexiganan, omiganan, novexatin, thionins, and thioneinetc) (Salas et al., 2015; Molchanova et al., 2017; Gründemann et al., 2019). Plants are a promising source of AMPs and a number of these peptides have been identified from different parts of plant (leaves, roots, seeds, flowers, and stems) that demonstrated significant activity against both human pathogen or phytopathogens (Montesinos, 2007; Benko-Iseppon et al., 2010; Nawrot et al., 2014). Being discovered from plant, they might have possible link with their ethno-medicinal uses against infection or other ailment.

The Solanaceae is an important family both for economic plants and medicinal plants. Potato, tomato, eggplant, and peppers are some of the most important cash crops that belong to the family of Solanaceae (Ghatak et al., 2017). On the other hand Atropa, Hyoscymus, Withania, Capsicum, and Nicotiana are just some of the most important Solanaceae plants that dictated early stages of medicinal plant based drug discovery and still considered important in herbal practice (Chowanski et al., 2016). The Solanaceae family consists of about 2,700 species distributed in 98 genera (Olmstead and Bohs, 2006). The Solanaceae is a family of flowering plants that ranges from annual and perennial herbs to vines, shrubs, and trees with their distribution in (Nath et al., 2017) almost all continents except Antarctica (Yadav et al., 2016). The Solanaceae are rich in alkaloids some of which finds their use in different traditional medicinal systems including Ayurveda, Traditional Chinese Medicine (TCM), Siddha, Unani, and homeopathy (Shah et al., 2013; Chowanski et al., 2016) especially for their use as antimicrobial, insecticidal, antiinfectious agents, and as poisons (Niño et al., 2006; Shah et al., 2013; Chowanski et al., 2016; Tamokou et al., 2017). Bioactive secondary metabolites reported from the members of the Solanaceae include AMPs, alkaloids, flavonoids, glycosides, lactones, lignans, steroids, simple phenols, sugars, and terpenoids (Ghatak et al., 2017). AMPs of plant origins act as chemical shields to protect plants from organisms and pests that directs to an interesting prospect of AMPs for possible use as promising molecules in antiinfective therapy (Campos et al., 2018). Literature study showed that a number of bioactive AMPs have been reported from different plant parts of the Solanaceae which confirmed the presence of such molecule in this family (Segura et al., 1999; Ryan and Pearce, 2003; Poth et al., 2012; Meneguetti et al., 2017; Kaewklom et al., 2018). However, there is no focused review of AMPs from plants of the Solanaceae to-date, despite their potential as natural antibiotics or antimicrobial agents. The aim of this review is to summarize the reported AMPs from plants of Solanaceae and to draw a possible molecular mechanism of action to further correlate the traditional uses of these plants with their reported AMPs.

### Search Strategy and Data Extraction

**Figure 1:**
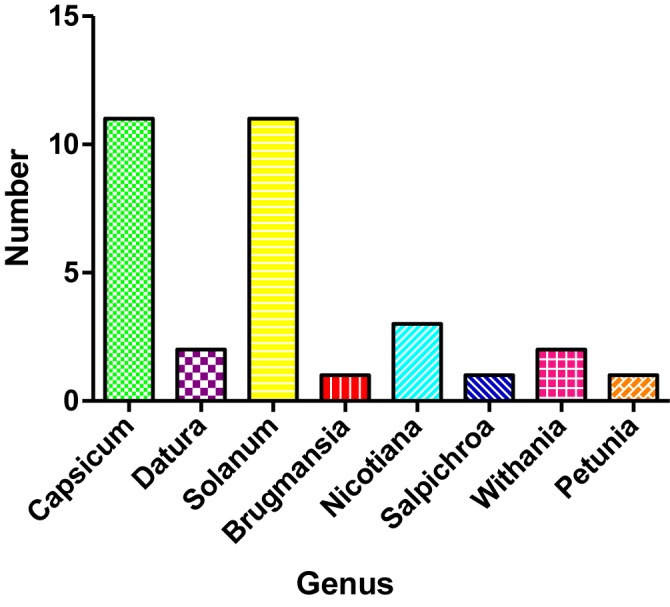
Reported antimicrobial peptides (AMPs) from different genus of Solanaceae family.

In this review, a comprehensive literature search was conducted using Google Scholar, PubMed, Science Direct, Scopus and Web of Science databases with the term “Solanaceae” along with “peptide,” “protein,” “AMP,” “antimicrobial,” “antifungal,” “antibacterial,” and “antiviral.” We have considered the reports that were only in English because of language barrier, time efficiency and nonfeasible costs of translation. Criteria for inclusion of investigation in this review: (a) peptides isolated from the plants of the Solanaceae, (b) studies those include the antimicrobial effects of peptide or peptide extract from the Solanaceae, (c) studies with peptide concentrations or doses employed, (d) studies of isolated peptides mass and sequence, (e) studies with mechanisms of action associated with their isolated peptides or peptide rich extracts. For the data extraction, all the retrieved articles were assessed according to surname of first author, publication year, the Solanaceae plants, peptides isolated and their mass, sequences, antimicrobial activity, concentrations used, and molecular mechanism involved. From the literature search, it was found that among all the genera of the Solanaceae, *Capsicum* and *Solanum* genera are more abundant with AMPs (**Figure 1**).

### AMPs From Plants of the Solanaceae Family

AMPs from plants are considered as barrier defensive chemicals that have protective response to predators like bacteria, fungi, nematodes, insects, and pests (Nawrot et al., 2014). Based on features, AMPs are grouped into different classes such as type of charge, disulfide bonds present, cyclic structure and the mechanism of action. Cyclotide, defensins, hevein-like proteins, knotin-type proteins, lipid transfer proteins, protease inhibitor, snakins, and thionins were the common classes of AMPs reported so far (Kim et al., 2009; Campos et al., 2018). Among these peptides defensins, protease inhibitor, lectins, thionin-like peptide, vicilin-like peptide, snaking, and some other AMPs were isolated and identified from Solanaceae. Isolated peptides and peptide rich extracts of plants from the Solanaceae exerted antimicrobial activity against various strains of bacteria, fungi, and viruses. **Tables 1** and **2** summarize the antimicrobial activity of peptide rich extract and isolated peptides from Solanaceae.

**Table 1:** Antimicrobial activity of peptide rich plants extract from Solanaceae family.

Genus	Plant name	Protein/Peptide (Class/Name)	Mass (kDa)	Sequence	Activity	MIC/MBC/IC_50_	Microorganism	Mechanism of action	Ref.
Capsicum	*Capsicum annuum* L.	Peptide rich extracts	5–12	NA	Antifungal	50 μg/ml	*C. gloeosporioides*	Inhibits the growth and hyphae formation	(Maracahipes et al., 2019)
		CWE1 peptide- extracts (leaf)	10	NA	Antibacterial	10 µg/ml 20 µg/ml 17.4 μg/ml	*R. solanacearum, C. michiganensis E. carotovora ssp*	NA	(Games et al., 2013)
					Antifungal	NA	*A. solani*		
	*Capsicum baccatum var. pendulum* (Willd.) Eshbaugh	Trypsin inhibitors rich leaf extract	10–14	Cb1= GFPFLLNGPDQDQGDFIMFG Cb-1′= GFKGEQGVPQEMQNEQATIP	Antiviral	1 μg/ml	*Pepper yellow*	Inhibits the activity of pathogen-derived proteinase by binding to and, thus, blocking its active site, suppressing enzymatic activity	(Moulin et al., 2014)
	*Capsicum frutescens* L.	Antimicrobial peptide rich leaf and fruit extract	NA	NA	Antibacterial	250 mg/ml	*E coli* *S. aureus* *K. pneumonia*	NA	(Dev and Venu, 2016)
					Antifungal	5 mg/ml	*Alternaria, Colletotrichum Fusarium*		
Datura	*Datura stramonium* L.		9–45	NA	Antibacterial	NA	*E. coli* *K. pneumoniae*	Binds to GlcNAc (N-acetyl glucosamine) oligomers which is responsible for the bacterial recognition.	(Muhammad et al., 2019)
Solanum .	*Solanum marginatum L*.	Protein rich extract (leaves)	18–112	NA	Antibacterial	0.1–10 µg/ml	*E. coli* *S. aureus*, *P. aeruginosa* *S. choleraesuis*	NA	(Guzmán-Ceferino et al., 2019)
	*Solanum stramonifolium Jacq*.	Protease inhibitors rich extracts (seed)	10– 21.5	NA	Antibacterial	100 µg/disc	*S. aureus* *B. licheniformis* *B. subtilis* *X. sp*. *P. aeruginosa* *S. typhi*	NA	(Sarnthima and Khammuang, 2012)

E. coli, Escherichia coli; K. pneumonia, Klebsiella pneumonia; S. aureus, Stapyllococcus aureus; B. licheniformis, Bacillus licheniformis; B. subtilis, Bacillus subtilis; P. aeruginosa, Pseudomonas aeruginosa; S. typhi, Salmonella typhi; S. choleraesuis, Salmonella choleraesuis; C. gloeosporioides, Colletotrichum gloeosporioides; R. solanacearum, Ralstonia solanacearum; C. michiganensis, Clavibacter michiganensis; E. carotovora ssp, Erwinia. carotovora ssp; A. solani, Alternaria solani; A. Colletotrichum, Alternaria Colletotrichum.

**Table 2:** Antimicrobial activity of isolated peptides from plants of Solanaceae family.

Genus	Plant name	Protein/Peptide (Class/Name)	Mass (kDa)	Sequence	Activity	MIC/MBC/IC_50_	Microorganism	Mechanism of action	Ref.
Brugmansia	*Brugmansia x candida* Pers.	Defensin	5.29	FSGGDCRGLRRRCFCTR-NH2	Antibacterial	15.70 μM	*E. coli* *V. cholerae* *S. sonnei* *S. typhimurium* *E. faecalis* *B. cereus* *S. epidermidis*	Affects cell membrane potential and permeability, and causes cell membrane disruption	(Kaewklom et al., 2018)
Capsicum	*Capsicum annuum* L.	Trypsin inhibitor	~ 20	NA	Antifungal	64 μg/ml	*F. solani* *C. gloeosporioides C. lindemuthianum F. oxysporum*	Causes hyphal morph–ological alterations, membrane permeabili- -zation *via* induces reactive oxygen species.	(Silva et al., 2017)
		Thionin-like peptide	5	NA	Antifungal	10 μg/ml, 20 μg/ml 40 μg/ml	*Candida* species	Causes plasma membrane permeabilization in all yeasts tested and induces oxidative stresses only in Candida tropicalis	(Taveira et al., 2016)
		Thionin-like peptides	7–10	NA	Antibacterial	100 µg/ml	*P. aeruginosa* *E. coli*	Induces change in the membranes of all strains, leading to their permeabilization	(Taveira et al., 2014)
					Antifungal	100 µg/ml	*S. cerevisiae* *C. albicans* *C. tropicalis*		
		Antimicrobial CaAMP1 protein	21.152	NA	Antibacterial	10 µg/ml, >100 µg/ml	*B. subtilis* *M. luteus*	NA	(Lee et al., 2008)
					Antifungal	30 µg/ml, 20 µg/ml, 5 µg/ml, 10 µg/ml, 5 µg/ml, >100 µg/ml, 50 µg/ml, 50 µg/ml	*C. albicans* *B. cinerea* *C. cucumerinum* *P. capsici* *S. cerevisiae*, *R. solani* *A. brassicicola* *F. oxysporum*	Inhibition of fungal spore germination and hyphae growth	
	*Capsicum baccatum* L.	Vicilin-like peptides	4–8	NA	Antifungal	200 µg/ml	*S. cerevisiae* *C. albicans* *C. tropicalis* *K. marxiannus*	Promotes morpholo-logical changes in all strains, including pseudohyphae formation	(Bard et al., 2014)
	*Capsicum chinense* Jacq.	Trypsin -chymotrypsin protease inhibitor	5.0–14	PEF2-A= QICTNCCAGRKGCNYYSAD PEF2-B= GICTNCCAGRKGCNYFSAD	Antifungal	100 µg/ml	*C. albicans*, *P. membranifaciens* *S. cerevisiae* *C. tropicalis* *K. marxiannus*	Exhibits cellular agglomeration and formation of pseudohyphae	(Dias et al., 2013)
		DING Peptide	7.57 And 39	~ 7.57 kDa =lengths of 32 (AGTNAVDLSVDQLCGVTSGRITTWNQLPATGR), 21 (ITYMSPDYAAPTLAGLDDATK), and 12 (RSASGTTELFTR) ~ 39 kDa= ITYMSPDYAAPTLAGLDDATK	Antifungal	3.75 µg/ml	*S. cerevisiae*	NA	(Brito-Argáez et al., 2016)
Datura	*Datura innoxia* Mill.	Chito-specific Lectin	9	NA	Antibacterial	0.325 mg/ml 0.25 mg/ml 0.15 mg/ml 0.5 mg/ml	*S. aureus* *B. cereus* *E. faecalis* *E. coli* *S. typhimurium* *P. aeruginosa*	NA	(Singh and Suresh, 2016)
					Antifungal	NA	*C. albicans* *T. viride* *G. saubinetii* *F. oxysporum* *C. sp* *S. cerevisiae* *F. moniliforme* *A. sfalvus*		
Nicotiana	*Nicotiana alata* Link & Otto.	Defensin (class I NaD1 and II NaD2)	11.72	MARSLCFMAF AILAMMLFVA YEVQARECKT ESNTFPGICI TKPPCRKACI SEKFTDGHCS KILRRCLCTK PCVFDEKMTK TGAEILAEEA KTLAAALLEE EIMDN	Antifungal	NaD1= 1μM, 0.5 μM, 0.75 μM, 1 μM, 0.8 μM, 2.5 μM, 2 μM NaD2= 5 μM, 2μM, >10 μM, 7 μM, 5 μM, 4 μM, 5 μM	*F. oxysporum* *F. graminearum* *V. dahlia* *T. basicola* *A. nidulans* *P. coronate* *P. sorghi*	Inhibits germination, stunting of germ tubes and a granular appearance of the cytoplasm in spores, reduces pustule frequency and increased photosynthetic area	(Dracatos et al., 2014)
		Defensin	5–7		Antifungal	10 µg/ml 2 µg/ml	*B. cinerea* *F. oxysporum*	Inhibits the hyphal growth	(Lay et al., 2003)
	*Nicotiana tabacum* L.	CBP20 Peptide	20	(CBP-PEP1): Y(A/G)SPSQGXQSQ(R)SGGGGGGGGGGGGGAGN (CBP-PEP2): TAFYGPVGP(P/R)GRDSXGK(G)	Antifungal	6.7 µg/ml	*F. solani* *T. viride* *A. radicina*	Causes lysis of the germ tubes	(Ponstein et al., 1994)
Petunia	*Petunia violacea var. hybrida* ****Hook. (syn. *Petunia hybrida* Vilm.)	Defensin	5 -7	NA	Antifungal	10 µg/ml 2 µg/ml	*B. cinerea* *F. oxysporum*	Inhibits the hyphal growth	(Lay et al., 2003)
Solanum	*Solanum lycopersicum* L.	Defensin	5.3–8.7	NA	Antifungal	2.5 µg/ml	*B. cinerea*	Inhibits hyphal tip growth	(Stotz et al., 2009)
		Snakin-2 peptide	7.05	NA	Antibacterial	4.25 µM 1.06 µM .26 µM 1.06 µM	*E. coli*, *A. tumefaciens* *M. luteus* *S. cohnii*	Perforates the biomembranes of bacteria and fungi	(Herbel et al., 2015)
					Antifungal	8.49 µM 4.25 µM	*P. pastoris*, *F. solani*		
	*Solanum tuberosum* L. cv Jaerla	Snakin-2 peptide	7.02	NA	Antibacterial	1 µM 30 µM 8 µM	*C. michiganensis* *R. solanacearum* *R. meliloti*	Induces rapid aggregation of both gm(+) and gm (−) bacteria	(Berrocal-Lobo et al., 2002)
					Antifungal	2 µM 3 µM 2 µM 10 µM 20 µM 10 µM 10 µM 10 µM 20 µM	*B. cinerea* *F. solani* *F. culmorum* *F. oxysporum* *A. flavus* *C. graminicola* *P. cucumerina* *C. lagenarium* *B. maydis*	NA	
	*Solanum aethiopicum* L. (syn. *Solanum integrifolium* Poir.)	Chitin-binding lectin	16.8	MKTIQGQSATTALTMEVARVQA	Antifungal	1 mg/ml 5 mg/ml	*R. solani* *C. gloeosporioides*	Inhibits the rate of the growth of fungal hyphae	(Chen et al., 2018)
					Insecticidal	1 μg/ml	*sf21 insect cells*	Reduces the mitochondrial membrane potential in insect cells	
	*Solanum tuberosum* L.	Serine protease inhibitor	13.5	NH2-LPSDATLVLDQTGKELDARL	Antifungal	6.25 µg/ml 6.25 µg/ml 6.25 µg/ml >100 µg/ml >100 µg/ml >100 µg/ml	*S. cerevisiae* *T. beigelii* *C. albicans* *C. gloeosporioides C. coccodes* *D. bryoniae*	NA	(Park et al., 2005)
		Trypsin-chymotrypsin protease inhibitor	5.6	NH2-DICTCCAGTKGCNTTSANGAFICEGQSDPKKPKACPLNCDPHIAYA	Antibacterial	50 µM	*C. michiganense*	Inhibits the growth of both types of microorganism.	(Kim et al., 2005)
					Antifungal	100 µM	*C. albicans* *R. solani*		
		Apoplastic hydrophobic peptides (AHPs)	12–78	NA	Antifungal	25 µM	*P. infestans*	Inhibits the germination of hyphae and accelerates the destruction of fungal spores	(Fernández et al., 2012)
		Potide-G	5.57	NA	Antiviral	90 µM	*P. Virus*	NA	(Tripathi et al., 2006)
							
Salpichroa	*Salpichroa origanifolia* (Lam.) Baill.	Aspartic protease inhibitor	32	NA	Antifungal	1.2 µM	*F. solani*	Causes permeabilization of cell membranes	(Díaz et al., 2018)
					Antibacterial	1.9 µM 2.5 µM	*E. coli* *S. aureus*		
Withania	*Withania somnifera* L. Dunal.	Lectin-like peptide	30	NA	Antifungal	7 μg/ml 9 μg/ml 11 μg/ml	*T. vesiculosum* *F. moniliforme* *M. phaseolina* *R. solani*	Inhibits the hyphal extension	(Ghosh, 2009)
		Glycoprotein (WSG)	28	NA	Antibacterial	20 µg/ml	*C. michiganensis*	Inhibits bacterial growth	(Girish et al., 2006)
					Antifungal		*A. flavus* *F. oxysporum*, *F. verticilloides*	Exerts a fungistastic effect by inhibiting spore germination and hyphal growth	

A. brassicicola, Alternaria brassicicola; A. tumefaciens, Agrobacteriumtumefaciens; A. radicina, Alternaria radicina; A. flavus, Aspergillus flavus; B. cinereal, Botrytis cinerea; B. subtilis, Bacillus subtilis; B. graminis, Blumeria graminis; B. cinerea, Botrytis cinerea; B. cereus, Bacillus cereus; B. cereus, Bacillus cereus; C. sp, Cephalosporium sp; C. cucumerinum, Cladosporiumcucumerinum; C. albicans, Candida albicans; C. tropicalis, Candida tropicalis; C.michiganensis, Clvibacter michiganensis; C. gloeosporioides, Colletotrichum gloeosporioides; C. occodes, Colletotrichum coccodes; C. michiganense, Clavibacter michiganense; C. lindemuthianum, Colletotrichum lindemuthianum; C. tropicalis, Candida tropicalis; D. bryoniae, Didymella bryoniae; E. faecalis, Enterococcus faecalis; E. coli, Escherichia coli; F. solani, Fusarium solani; F. graminearum, Fusariumgraminearum; F.moniliforme, Fusariummoniliforme; F. oxysporum, Fusariumoxysporum; F. verticilloides, Fusariumverticilloides; G. saubinetii, Gibberella saubinetii; K. marxiannus, Kluyveromyces marxiannus; M. phaseolina, Macrophomia phaseolina; M. luteus, Micrococcus luteus; P. infestans, Phytophthora infestans; P. Virus, Potato Virus; P. graminis, Puccinia graminis; P. capsica, Phytophthora capsici; P. triticina, Puccinia triticina; P. hordei, Puccinia hordei; P. striiformis, Puccinia striiformis; P. coronate, Puccinia coronate; P. aeruginosa, Pseudomonas aeruginosa; P. nodorum, Phaeosphaeria nodorum; R. solani, Rhizoctonia solani; R.meliloti, Rhizobiummeliloti; S. sonnei, Shigella sonnei; S. typhimurium, Salmonella typhimurium; S. epidermidis, Staphylococcus epidermidis; S. cerevisiae, Saccharomyces cerevisiae; S. aureus, Staphylococcus aureus; S. cohnii, Staphylococcus cohnii; T. vesiculosum, Trichosporium vesiculosum; T. viride, Trichoderma viride; T. controversa, Tilletia controversa; T. beigelii, Trichosporon beigelii; U. tritici, Ustilago tritici; V. cholera, Vibrio cholera; NA, Not available.

Several genera of the Solanaceae, such as *Capsicum, Datura*, and *Solanum*, have been reported to possess AMPs and peptide rich extract from seeds, leaf or fruit, tuber of these species. These peptides have been reported to have significant antibacterial, antifungal, or antiviral activities against both phytopathogenic and human pathogenic strain (**Table 1**). The reported AMP rich extracts belong to different categories include acidic, basic, protease inhibitor, and trypsin inhibitors (Sarnthima and Khammuang, 2012; Moulin et al., 2014; Muhammad et al., 2019). The mechanism of their action was not clear, however, it was reported that antibacterial activity could be due to changes in membrane permeabilization (Muhammad et al., 2019) and antifungal activity could be owing to inhibition of fungal growth and hyphae formation (Maracahipes et al., 2019). The *Datura* is a common genus of the Solanaceae and mostly found in Asian continent with a number of ethnomedicinal uses including against microbial infections (**Table 3**). Recently, Muhammad et al. (2019) reported that the seed extract of *Datura stramonium* L. is rich in acidic and basic peptides (9–45 kDa) and exhibited antibacterial activity against *Escherichia coli* and *Klebsiella pneumonia* (Eftekhar et al., 2005; Muhammad et al., 2019). Antibacterial activity of peptide rich extract from the leaves of *Solanum stramonifolium* Jacq. and seeds of *Solanum marginatum* L.f. showed antibacterial activity against different human pathogenic bacteria with the MIC values 0.1–100 µg/ml (Sarnthima and Khammuang, 2012; Guzmán-Ceferino et al., 2019). Peptide rich leaf and seed extracts of different species of the *Capsicum*, e.g., *Capsicum annuum* L. and *Capsicum frutescens* L., exhibited significant antibacterial and antifungal effect *via* inhibiting their growth and hyphae formation (Games et al., 2013; Dev and Venu, 2016; Maracahipes et al., 2019). A study by Moulin et al. (2014) showed that trypsin inhibitors (10–14 kDa) rich leaf extract of *Capsicum baccatum var. pendulum* (Willd.) Eshbaugh exerted antiviral activity (MIC 1–25 µg/ml) against PepYMV (Pepper yellow mosaic virus) by blocking the active site of pathogen-derived proteinase as well as reduced enzymatic activity (Moulin et al., 2014). The genera *Capsicum*, *Datura*, and *Solanum* of the Solanaceae are popular in ethnobotany and have been reported to have different traditional uses against different diseases including infections (**Table 3**) which might be linked to the AMPs found in these plants.

**Table 3:** Traditional uses of plants from Solanaceae family.

Plant name	Traditional uses	References
*Brugmansia x candida* Pers.	Used as analgesic against traumatic or rheumatic pains as well as for the treatment of dermatitis, orchitis, arthritis, headaches, infections, and as an antiinflammatory.	(Feo, 2004)
*Capsicum annuum* L.	Used to prevent cold, sinus infection, sorethroat and improve digestion, blood circulation, cancer, asthma, and cough, norexia, haemor-rhoids, liver congestion, and varicose veins.	(Duke, 1993; Khare, 2004)
*Capsicum baccatum* L.	Antirheumatic, antiseptic, diaphoretic, digestive, irritant, rubefacient, sialagogue and tonic	(Bown, 1995; Chevallier, 1996)
*Capsicum chinense* Jacq	Asthma, gastro-intestinal abnormalities, toothache and muscle pain, removal of puss from boils, arthritis	(Roy, 2016)
*Capsicum frutescens* L.	Antihaemorrhoidal, antirheumatic, antiseptic, carminative, diaphoretic, digestive, sialagogue and stomachic, antibiotic properties.	(Chiej, 1984; Simpson and Conner-Ogorzaly, 1986; Chevallier, 1996)
*Datura stramonium* L.	Used to treat epilepsy burns and rheumatism, anthelmintic, and antiinflammatory, worm infestation, toothache, and fever, insect repellant, which protects neighboring plants from insects.	(Guarrera, 1999; Das et al., 2012; Soni et al., 2012)
*Datura innoxia* Mill.	Used in the treatment of insanity, fevers with catarrh, diarrhea, and skin diseases.	(Chopra and Chopra, 1969; Emboden, 1972)
*Nicotiana alata* Link & Otto.	Used as antiseptic, insecticide, antispasmodic, relieve pain, and swelling associated with rheumatic conditions and vermifuge.	(Binorkar and Jani, 2012)
*Solanum lycopersicum* L.	First aid treatment for burns, scalds and sunburn, treatment of toothache	(Duke, 2008)
*Solanum tuberosum* L.	Folk remedy for burns, corns, cough, cystitis, fistula, prostatitis, scurvy, spasms, tumors, and warts	(Duke and Wain, 1981; Graham et al., 2000)
*Salpichroa origanifolia* (Lam.) Baill.	Used as antiinflammatory, diuretic, antimicrobial and narcotic effect	(Parisi et al., 2018)
*Withania somnifera* (L.) Dunal.	Aphrodisiac, sedative, chronic fatigue, weakness, dehydration, weakness of bones and loose teeth, thirst, impotence, premature aging, emaciation, debility and muscles tension, antihelmantic.	(Mir et al., 2012)

**Figure 2:**
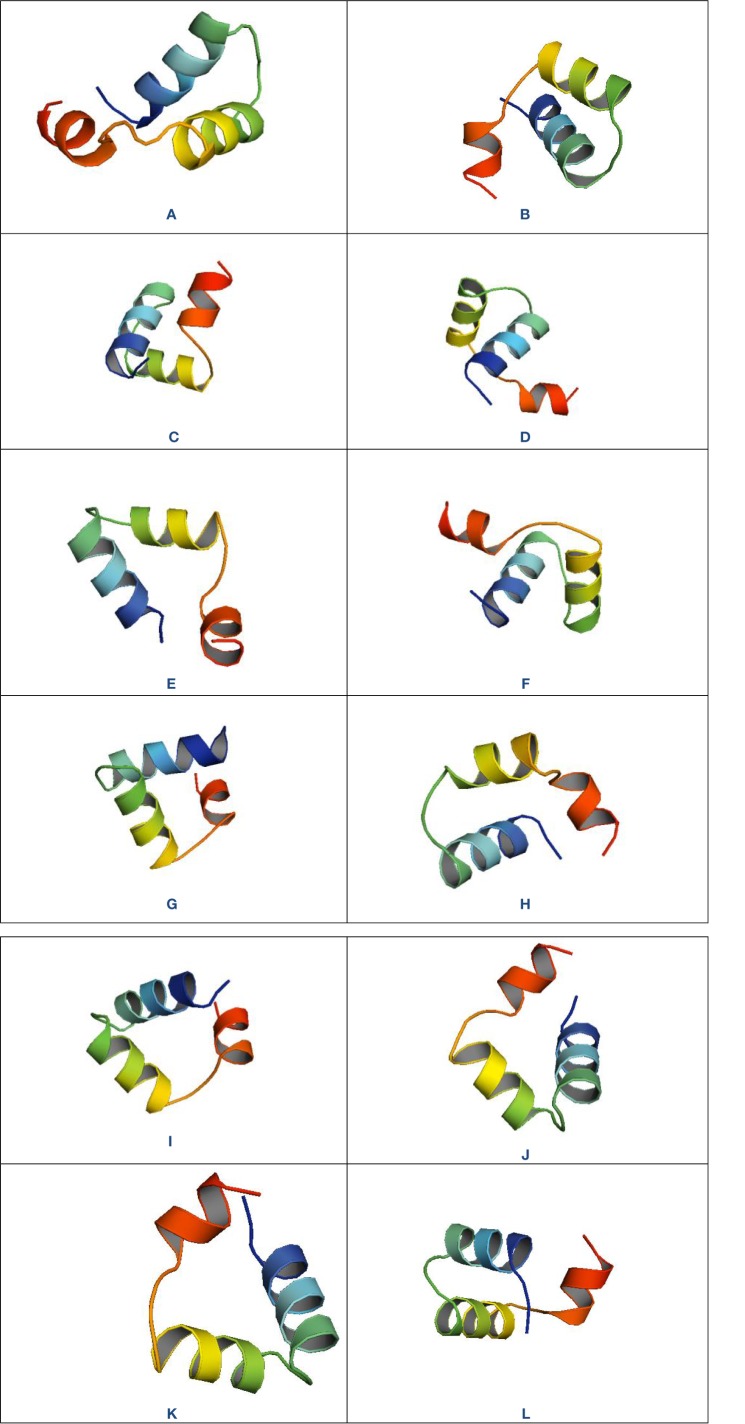
3D structures of different antimicrobial peptides (AMPs) of the Solanaceae family. “PEPFOLD 3.5 De Novo Peptide Structure Prediction” program from “RPBS Web Portal” (https://mobyle.rpbs.univ-paris-diderot.fr/) was used to draw the 3D structures. The program was executed with highest number of simulations (200) and 3D models were sorted by sOPEP. The best models were downloaded and opened with PyMOL(TM) 2.3.2 - Incentive Product, Copyright (C) Schrodinger, LLC and the structures were captured ensuring publication quality. **(A)** Defensin from *Brugmansia x candida* (FSGGDCRGLRRRCFCTR-NH2); **(B)** Trypsin inhibitor from *Capsicum baccatum var. pendulum* (Cb1=GFPFLLNGPDQDQGDFIMFG); **(C)** Trypsin inhibitor from *Capsicum baccatum var. pendulum* (Cb1) (GFKGEQGVPQEMQNEQATIP); **(D)** Trypsin-chymotrypsin protease inhibitor from *Capsicum chinense* (PEF2-A) (QICTNCCAGRKGCNYYSAD); **(E)** Trypsin -chymotrypsin protease inhibitor from *Capsicum chinense* (PEF2-B) (GICTNCCAGRKGCNYFSAD); **(F)** DING peptide from *Capsicum chinense* (AGTNAVDLSVDQLCGVTSGRITTWNQLPATGR)]; **(G)** DING peptide from *Capsicum chinense* (RSASGTTELFTR)]; **(H)** DING peptide from *Capsicum chinense* (ITYMSPDYAAPTLAGLDDATK); **(I)** Defensin (NaD1 and NaD2) from *Nicotiana alata* (MARSLCFMAFAILAMMLFVAYEVQARECKTESNTFPGICITKPPCRKACISEKFT DGHCSKILRRCLCTKPCVFDEKMTKTGAEILAEEAKTLAAALLEEEIMDN); **(J)** Serine protease inhibitor from *Solanum tuberosum* (NH2-LPSDATLVLDQTGKELDARL); **(K)** Trypsin-chymotrypsin protease inhibitor from *Solanum tuberosum* (NH2-DICTCCAGTKGCNTTSANGAFICEGQSDPKKPKACPLNCDPHIAYA); **(L)** Chitin-binding lectin from *Solanum integrifolium* (MKTIQGQSATTALTMEVARVQA).

Plant defensins are cysteine rich small (45 to 54 amino acids) basic peptides that can form four structure-stabilizing disulfide bridges (Benko-Iseppon et al., 2010). They have a widespread distribution and are likely to be present in the Solanaceae. Kaewklom et al. (2018) reported a new plant defensin (5.29 kDa) with interesting structural and biological features from *Brugmansia x candida* Pers. that showed antibacterial activity (MIC of 15.7 μM) against *Bacillus cereus*, *Enterococcus faecalis, E. coli, Shigella sonnei, Salmonella typhimurium, Staphylococcus epidermidis*, and *Vibrio cholerae*, by affecting membrane permeability, membrane potential, and membrane disruption (Kaewklom et al., 2018). Different types of defensin were found in *Nicotiana alata* Link & Otto that inhibit germination and the hyphal growth of fungus (Lay et al., 2003; Dracatos et al., 2014) (**Figure 2**). Antifungal defensins were also found from *Solanum lycopersicum* L. and *Petunia violacea var. hybrida* Hook. (syn. Petunia hybrida Vilm.) with MICs of 2.5–11 µg/ml against *Botrytis cinerea* and *Fusarium oxysporum* through inhibition of hyphal tip growth (Stotz et al., 2009). Interestingly, *B. x candida*, *N. alata*, *S. lycopersicum*, and *P. hybrida* have long been used traditionally for treating various diseases which is justified by the defensin content of these plant species of Solanaceae.

Proteinase inhibitors are another class of plant peptides that reported to possesses antibacterial and antifungal activity (Hancock and Lehrer, 1998; Epand and Vogel, 1999; Kim et al., 2009). Plant protease inhibitors are commonly found in tubers and seeds and known to inhibit aspartic, cysteine, and serine proteinases. Increased levels of trypsin and chymotrypsin inhibitors in plants have a strong correlation with their resistance to the pathogen (Kim et al., 2009). *Solanum tuberosum* L. is a common species of the Solanaceae and different protease inhibitor-like AMPs have been reported from this species. Park et al. (2005) and Kim et al. (2005) reported trypsin-chymotrypsin and serine protease inhibitor-like peptides from *Solanum tuberosum* and both demonstrated potential antifungal activity with MICs 1–25 µg/ml (Kim et al., 2005; Park et al., 2005). Among these peptides, iskunitz-type serine protease inhibitor was reported to be active against *Candida albicans, Colletotrichum gloeosporioides, Colletotrichum coccodes, Didymella bryoniae, Saccharomyces cerevisiae*, and *Trichosporon beigelii* fungal infections whereas the other one trypsin-chymotrypsin protease inhibitor was active against *C. albicans* and *Rhizoctonia solani*. The genus *Capsicum* produces trypsin and trypsin-chymotrypsin protease inhibitor like peptides with antifungal activity (MIC 50-250 µg/ml), particularly from *C. annuum* and *C. chinense* Jacq. (Dias et al., 2013; Silva et al., 2017). The antifungal activity of these AMPs exhibited either through cellular agglomeration and formation of pseudohyphae or *via* hyphal morphological alterations as well as membrane permeabilization by inducing ROS (Dias et al., 2013; Silva et al., 2017). *Salpichroa origanifolia* is another plant of the Solanaceae from which another aspartic protease inhibitor AMP has been reported that possesses both antifungal (0.3–3.75 µM) and antibacterial (0.32.5 µM) activity against *Fusarium solani*, *E. coli*, and *Staphylococcus aureus via* membrane permeabilization (Díaz et al., 2018). Interestingly, *Capsicum*, *Salpichroa*, and *Solanum* are well known genera of the Solanaceae and have been used in traditional medicine against a number of infectious diseases (**Table 3**).

Lectins are carbohydrate binding proteins, widely distributed in plants, animals, or microorganisms and have specificity for cell surface sugar moieties of glycoconjugates residues (Brooks and Leathem, 1998). Plant lectins have been reported to a wide variety of flowering plant species (Allen and Brilliantine, 1969). The Solanaceae is a family of flowering plants and a number of lectins have been reported from different plants from this family (**Table 2**). Antimicrobial action of lectins has long been known and the reported lectins from the Solanaceae also possess antibacterial and antifungal activity. A chito-specific lectin (9 kDa) was purified and characterized from *Datura innoxia* Mill. seeds that was shown to have antibacterial and antifungal activity at different concentrations against various strains of bacteria (MICs 0.25–0.5 mg/ml) and fungi (MIC 0.15 mg/ml) (Singh and Suresh, 2016). Lectin-like protein (30 kDa) was isolated from *Withania somnifera* (L.) Dunal that showed antimicrobial effect (MIC 7-11 μg/ml) (Girish et al., 2006; Ghosh, 2009). Recently, Chen et al. (2018), reported a chitin-specific lectin from *Solanum aethiopicum* L. (syn. *Solanum integrifolium*) with antifungal (MIC 1–5 mg/ml) and insecticidal activities (MIC 1 μg/ml) (Chen et al., 2018). Another monomeric glycoprotein (28 kDa) was reported from *W. somnifera* root tubers which showed significant antimicrobial activity against phytopathogns (both fungi and bacteria) (Girish et al., 2006). The antifungal activity of reported lectins were due to the inhibition of growth and extension of fungal hypha (Girish et al., 2006; Ghosh, 2009; Chen et al., 2018). These plants have been reported to have traditional uses against different infections (**Table 3**) which might have correlation with the reported AMPs from these plants.

Thionins are another AMPs that are structurally cystine-rich, disulfide bond containing cationic small peptides (∼5 kDa) found in plant and act as a part of plant defense mechanisms (Westermann and Craik, 2010). It is reported that thionins possess cidal effect to a broad range of bacteria and mammalian cells through loss of membrane integrity and induces membrane permeabilization mechanisms (Montville and Kaiser, 1993; Westermann and Craik, 2010). Literature study demonstrated that *C. annuum* was a potential plant with thionins that showed antimicrobial activity against a broad ranges of human pathogens both bacteria (MIC 100–300 mg/ml) and fungi (MIC 10–40 µg/ml). The possible mechanism of action includes induced membrane permiabilization or changes in membrane integrity as well as induced oxidative stress (Taveira et al., 2014; Taveira et al., 2016). Interestingly, the *Capsicum* is one of the potential genera of the Solanaceae that has been used traditionally against a number of infectious diseases (**Table 3**).

Vicilins are 7S globulin class plant seed storage proteins with no disulfide bond and structurally contain three similar subunits of 40–70 kDa (Bard et al., 2014). These proteins possess different functions and known as plant defense proteins (Jain et al., 2016). Vicilin-like peptides have similar homology with vicilin and exhibited antimicrobial and antifungal activity (Ribeiro et al., 2007; Jain et al., 2016). *Capsicum baccatum* L. has been reported to produce vicilin-like peptides that showed promising antifungal activity (MIC 100–200 µg/ml) (Bard et al., 2014). The possible mechanism of their antifungal activity was not clear but highlighted that the antifungal action was due to promotion of cellular morphological changes including pseudohyphae formation through binding of chitin containing components of fungal cell wall (Bard et al., 2014).

Snakins are plant AMPs that have twelve conserved cysteine residues and play different roles in plant with the responses of both biotic and abiotic stress. These plant peptides have been reported to offer a number of activities including significant antibacterial activity and therefore have potential therapeutic and agricultural applications (Oliveira-Lima et al., 2017). The *Solanum* genus is rich in snakin-2 peptide that possesses significant antimicrobial activity. Herbel et al. (2015) revealed that recombinant snakin-2 (7.05 kDa) protein in *E. coli* from *Solanum lycopersicum* caused perforation of membranes of bacteria and fungi with MIC values 0.26–8.49 µM (Herbel et al., 2015). Another snakin-2 peptide (7.02 kDa) was isolated from potato tuber (*S. tuberosum* ) that showed promising activity against phytopathogenic bacteria (MICs 1–30 µM) and fungi (MIC 1–20 µM). The mechanism of action of snakins remains unclear, however the antibacterial activity was reported due to the rapid aggregation of bacterial cells (Berrocal-Lobo et al., 2002).

In addition to these common plant AMPs, some other peptides or polypeptides with significant antimicrobial activity have also been reported from plants of the Solanaceae (**Table 2**). Brito-Argáez et al. (2016) reported a ~7.57 kDa peptide with interesting antifungal (MIC 3–15 µg/ml) and antiproliferative activity from *C. chinense* seeds, which were further confirmed a proteolytic product belonging to a ~ 39 kDa DING protein (Brito-Argáez et al., 2016). DING protein is a class of ubiquitous protein (40 kDa) that possesses phosphatase and inhibition of carcinogenic cell growth activity (Bookland et al., 2012) (**Figure 2**). A study conducted by Ponstein et al. (1994) demonstrated the purification of a new pathogen and wound-inducible polypeptide (CBP20) from tobacco leaves (*Nicotiana tabacum*) with antifungal activity (Ponstein et al., 1994) (**Figure 2**). A number of apoplastic hydrophobic proteins (AHPs) with antifungal activity identified after differentially expressed by *Phytophthora infestans* infection to potato tuber (*S. tuberosum*) that help to protect potato against *P. infestans* infection (Fernández et al., 2012). Inhibition of germination of hyphae and fungal spore was the possible mechanism of AHPs’s antifungal activity (Fernández et al., 2012). In 2006, two antiviral peptides named potide-G and golden peptide were isolated separately from potato (*S. tuberosum* L.) that showed promising antiviral activity against potato virus YO (PVYO) (Tripathi et al., 2006). Another study with *C. annum* found a new antimicrobial protein CaAMP1 that exhibited promising activity against both different bacteria (MICs 5–30 µg/ml) and fungi (MICs 5–100 µg/ml). The antifungal activity was due to inhibition of spore germination and hyphae growth (Lee et al., 2008). Some other peptides belonging to different AMPs families such as defensins, thionin, protease inhibitor, hevein-type were also reported from *S. tuberosum*., *C. annuum*. and *Solanum esculentum* L. of the Solanaceae that showed no antibacterial activity (Guevara et al., 2001; Carrillo-Montes et al., 2014; Kovtun et al., 2018). *Solanum, Capsicum, Nicotiana*, and *Withania* were the most ethnobotanical genera of the Solanaceae that have different traditional uses against different diseases including antimicrobial activity (**Table 3**) which could have correlation with these reported plant defensive AMPs.

AMPs have been studied for several decades but understanding of their molecular mechanism is still unclear. However, it is evident that AMPs are plant defense peptides that act against pathogen (both bacteria and fungi) to protect themselves by interacting with their cell wall. AMPs can act through several mechanism depending on peptides structure, amino acid sequence, peptide-lipid ratio as well as properties of the interacting lipid membrane (Galdiero et al., 2013; Bechinger and Gorr, 2017). It is evident that interaction of peptides with cell membrane causes changes in peptide’s conformation and aggregation state that adapted by membrane lipid *via* alteration of their (lipid) conformation and packing structure (Bechinger and Gorr, 2017). Both Gram-positive and Gram-negative bacteria contain negatively charged surfaces on outer membrane (Gram-negative) or cell wall (Gram-positive) and therefore there was no basic mechanistic difference of AMPs acting on them. Furthermore, Gram-positive bacterial cell wall contain pores (40 to 80 nm) and several AMPs easily cross it to interact with target site (Malanovic and Lohner, 2016). Sani and Separovic (2016) proposed a number of membrane models (barrel-stave pore, toroidal pore and carpet model) associated with cationic AMPs-membrane interaction, membrane disruption and membrane permiability (Sani and Separovic, 2016). In case of Gram-negative bacteria, AMPs cross membrane through electrostatic interaction and charge-exchange mechanism with Ca^2+^ and Mg^2+^ bound to lipopolysaccharide and peptidoglycan (Schmidt and Wong, 2013; Anunthawan et al., 2015). The mechanism of antibacterial action of peptides from Solanaceae were due to the induction of membrane pores, alteration of cell membrane potential and permeability as well as cell aggregation which support the reported AMPs mechanism of action. Whereas, antifungal AMPs can specifically target fungi cell wall or cell membrane and ergosterol is the major component in fungal cell membranes which regulates permeability and fluidity (Silva et al., 2014; Rodrigues, 2018). AMPs also exert their antifungal activity by inhibition of *β-*glucan synthase resulting in destabilized cell wall and cell lysis (Matejuk et al., 2010). The alteration of hyphal growth by AMPs was due to inhibition of cell wall biosynthesis (Theis et al., 2003). Interestingly, reported Solanaceae AMP’s antifungal activity were supported by the molecular mechanism such as induction of cell membrane permiabilization, inhibition of germination, and alteration of hyphal growth.

## Conclusion

In this review, we have summarized the reported AMPs from plants of the Solanaceae and pointed out the possible molecular mechanisms to correlate the ethnobotanical uses with their antimicrobial action. These data demonstrated that a variety of AMPs have been isolated with significant antimicrobial activity from plants of the Solanaceae including defensins, protease inhibitor, lectins, thionin-like peptide, vicilin-like peptide, snaking, and others. *Capsicum, Solanum, Datura, Nicotiana, Withania, Salpichora, Brugmansia*, and *Petunia* are the most promising genera to produce different AMPs. Alteration of cell membrane potential and permeability as well as membrane pores induction and cell aggregation were the possible antibacterial mechanism of the reported peptides. On the other hand, the antifungal activity was due to induction of cell membrane permeabilization, inhibition of germination and alteration of hyphal growth. However, the mechanisms of action of the AMPs from Solanaceae were not any new pathway rather similar to other generic AMPs. The isolated and identified AMPs from the Solanaceae are a part of its defense mechanism and are therefore have strong correlation with their ethnobotanical virtues including antimicrobial, poisonous, insecticidal, and antiinfectious. The Solanaceae contain a variety of AMPs with promising antimicrobial activity that may be a potential source of lead for antimicrobial drug development. In addition to pharmaceutical uses, AMPs from Solanaceae can also be a good source for development of innovative approaches for plant protection in agriculture. Conferred disease resistance by AMPs might help us surmount losses in yield, quality and safety of agricultural products as well as molecular farming due to their disease resistance properties. Furthermore, new species from Solanaceae could be interesting to be explored for novel AMPs.

## Author Contributions

The review was designed by SU and written by SU, MA, SA, AA, and RR. JS, ET, SS, AA, and UG provided valuable guidance, revision, correction, and other insight into the work.

## Conflict of Interest

The authors declare that the research was conducted in the absence of any commercial or financial relationships that could be construed as a potential conflict of interest.
